# Seroepidemiology of maternally-derived antibody against Group B Streptococcus (GBS) in Mulago/Kawempe Hospitals Uganda - PROGRESS GBS

**DOI:** 10.12688/gatesopenres.13183.2

**Published:** 2020-11-13

**Authors:** Mary Kyohere, Hannah Georgia Davies, Philippa Musoke, Annettee Nakimuli, Valerie Tusubira, Hannington Baluku Tasimwa, Juliet Sendagala Nsimire, Paul Heath, Stephen Cose, Carol Baker, Kirsty Le Doare, Musa Sekikubo

**Affiliations:** 1 Makerere University - Johns Hopkins University (MUJHU) Research Collaboration, Kampala, Uganda; 2 Paediatric Infection and Immunology Institute of Infection and Immunity, St George’s, University of London, London, SW170RE, UK; 3 Department of Paediatrics and Child Health, Makerere University, College of Health Sciences, Kampala, 256, Uganda; 4 Department of Obstetrics and Gynaecology,, Makerere University, College of Health Sciences, Kampala, 256, Uganda; 5 Department of Medical Microbiology, Makerere University College of Health Sciences, Kampala, 256, Uganda; 6 Clinical Diagnostics Laboratories, MRC/UVRI/LSHTM, Entebbe, Uganda; 7 Immunology and Vaccines Research Unit, MRC/UVRI and LSHTM Uganda, Entebbe, Uganda; 8 University of Texas Health Science Center, McGovern Medical School, Houston, Texas, TX 77030, USA

**Keywords:** Group B Streptococcus, antibody, neonatal sepsis, Uganda, neonate, infant, sero-epidemiology, vaccine

## Abstract

**Background**: Group B *Streptococcus* (GBS) is a major contributor to the high burden of neonatal and young infant infectious disease in resource- limited settings. As disease protection during the first six months of life is provided via placental transfer of maternal antibodies, a maternal GBS vaccine may provide an effective strategy to reduce infectious death and disability. An efficacy study may be difficult because of the large sample size required and alternative approaches such as serocorrelates of protection based on natural antibody concentration are being considered. Such studies would need to be undertaken in high burden settings such as Uganda. We therefore aim to evaluate the feasibility and acceptability of a GBS sero-epidemiology study in Kampala, Uganda.

**Methods**: This is a prospective cohort and nested case-control study, conducted across two-centres with two entry points. A) consecutive women and their infants at birth, with collection of maternal swab, cord and maternal blood, and follow up by telephone until the infant is 3 months old; B) any infant under 3 months of age, presenting with signs of sepsis to any of the paediatric units, with collection of blood culture, cerebrospinal fluid and nasopharyngeal swabs. Any infants identified as having GBS disease (defined as GBS isolated from a normally sterile site) will be recruited and followed up for two years to assess their neurodevelopment. A nested qualitative study will investigate stakeholder (pregnant women and their families, healthcare workers and community leaders) opinions of sampling for such a study and understanding and potential uptake of vaccines in pregnancy.

**Discussion**: The primary aim is to determine anti-GBS antibody concentration in infants with GBS disease compared to healthy controls. Secondary outcomes include stillbirth and all-cause infection and acceptance of sample methods and vaccination. The findings will inform scalability and sustainability of the programme in Uganda.

## Introduction

The last ten years have seen major improvements in the number of deaths in children less than five years of age in many regions of Africa. However, the unacceptably high death rates amongst newborns have not declined at the same rate. Approximately one-third of these deaths are due to severe infection. Group B *Streptococcus* (GBS) meningitis and severe bloodstream infection are likely to be major contributors to this very high disease burden in Africa and other resource-limited settings. Globally, GBS colonization is estimated to be present in 21.7 million pregnant women. A 2015 estimate showed more than 319,000 infants <3 months of age with invasive GBS disease (iGBS), 90,000 infant deaths and more than 10,000 children with GBS meningitis related disability ^1^. 33,000 maternal deaths and 57,000 stillbirths were also ascribed to GBS disease ^1^.

Intrapartum antibiotic prophylaxis (IAP) can reduce the incidence of early onset disease (EOD) but has no impact on late onset disease (LOD) and only a limited impact on disease in pregnant women ^2^. In settings such as Uganda, IAP implementation is likely to be poor because of irregular attendance at antenatal care. In the UK and USA, GBS is the commonest cause of bacterial meningitis in children under 5 ^1^, with most GBS meningitis occuring after week one of life, hence the burden persists.

Although GBS-colonization is common, very few colonized infants subsequently develop iGBS (1-2%). With regards to titers of naturally occurring serotype-specific maternal antibody to GBS-capsular polysaccharide (CPS), studies have shown a correlation between high titres and reduced risk of disease in neonates ^3^. Baker and colleagues ^4^ initially characterized the association between iGBS in newborns and serotype (ST)-specific CPS antibody levels in 1976. Infants with EOD and LOD were found to have lower ST-specific CPS antibodies than controls in most subsequent studies. There is now consensus that these data suggest that maternal immunization may be an effective strategy for protection ^5^.

 Results from a meta-analysis comparing the proportions of cases and controls with antibody levels ≥2 ug/ml for serotypes III and Ia, showed iGBS was (OR=6.56 CI: 2.10–20.55) and (OR=2.38 CI: 1.20–4.70) times greater in infants whose mothers had antibody levels <2 ug/ml respectively ^6^. 1 ug/ml has also recently been proposed as a threshold for correlate of protection against ST Ia and III ^3^. Other larger studies, including a study from South Africa ^7^, where HIV prevalence is high, show much higher thresholds. Lack of available standardized reference sera and differences in methods make interpretation of these results difficult. A standardized approach to establish antibody concentration and function and its role in reducing neonatal disease is vital in achieving licensure of any future vaccines and is currently underway through funding from the Bill and Melinda Gates Foundation and collaborators involved in this proposal ^8^.

The likelihood of protecting mothers and their infants through vaccination in pregnancy is made desirable by the early onset of neonatal GBS disease, the shortfalls of prevention strategies using IAP and evidence suggesting maternal antibodies transmitted transplacentally may protect the young infant from invasive infection ^3^

To be considered for an effective vaccine are the various conserved surface proteins or the CPS. The immunogenicity and safety of CPS - protein conjugate vaccines have been demonstrated through several studies in non-pregnant and, more recently, in pregnant women ^9^.

The most advanced vaccines face a number of obstacles in moving into phase III clinical trials. First, iGBS in Europe and the United States is relatively rare so determining efficacy of a vaccine in settings such as these would require large numbers of infants to be recruited ^10^. Second, there are obstacles determining the concentration of antibody that is needed to protect the infant throughout the first three months of life since, at the moment there are no standards with which to interpret individual study results that are recognized internationally ^11^. An immune marker, measured using a validated assay, and established as a correlate of protection would markedly accelerate policy and licensure decisions. A commitment for effectiveness post-licensure to be established would be made in this case, similar to the approach that was used for meningococcal C and meningococcal B vaccines licensure ^12^.

Such serocorrelates of protection should be based within high burden settings, such as Uganda and analysed in women living with HIV, where antibody transfer may be impaired, compared to HIV-negative women. Thus, this study aims to evaluate the feasibility and acceptability of undertaking a large sero-epidemiology study of GBS in two urban hospitals in Kampala, Uganda.

## Methods

The PROGRESS *Group B Streptococcus* Study is a feasibility and nested case-control study of the first 6000 women to deliver at Kawempe Hospital and active surveillance for 12 months at Kawempe National Referral Hospital and Mulago National Referral Hospital for neonatal infection. 

### Sample size calculation

Cases: Based on previous Correlate of Protection (CoP) studies ^3, 7^, feasible correlates are those that give 80-90% risk reductions. In a case-control study, an 80% reduction is equal to an Odds Ratio (OR) of 0.2. Table 1 shows the precision around 0.2 for varying case sample sizes and proportion of controls with IgG levels above the potential CoP levels (“cuts”).

**Table 1.** Precision of CoP estimates according to different sample sizes.

Controls > cut	N=30 cases N=90 controls	N=90 N=270 controls	N=150 N=450 controls
70%	0.08-0.49	0.12-0.33	0.13-0.30
50%	0.07-0.57	0.11-0.37	0.13-0.32
30%	0.05-0.81	0.09-0.45	0.11-0.37

Therefore with 30 cases the 95% CI around an 80% reduction is 43% to 93%; and with 90 cases it is 63% to 89% if 50% of controls have levels above the cutoff.

The incidence of iGBS among infants < 3 months of age in Uganda is not known. A study from Mulago Hospital in 5 months during 2002 identified 7 cases of GBS disease in a cohort of 293 infants admitted with signs of sepsis (2% of all infection, 25% of positive cultures (2 EOD, 5 LOD)) ^13^. A more recent study from 2010 in Mbarara identified 80 infants with presumed infections over a 10 month period identified 1 case of early onset GBS infection (1% of infections, 50% of positive cultures) ^14^. No serotype data is available from either study. These are likely underestimates due to manual culture methods used and recruits were only those infants who presented to the hospital with signs of infection and we know that in the first weeks of life many infants are born and die at home.

Based on the most recent analysis of cost-effectiveness for a potential GBS vaccine in Uganda, Guinea Bissau, Nigeria and Ghana, GBS disease incidence of 1.13/1000 livebirths was used to demonstrate the prospects of a multivalent vaccine against GBS to prevent approx. 1/3 of all GBS cases and deaths in Uganda ^15^.

Following a cohort of 35000 women with an incidence of 1.13/1000 livebirths therefore would be expected to yield 40 cases (95% CI 33-46); with an incidence of 2/1000 we would expect 70 cases (95% CI 61-79) of iGBS per annum with a sufficient number of cases for at least one of the most frequent serotypes i.e. STIII and STIa.

Controls: Controls are infants who are exposed to the same ST / strain of GBS at delivery as the case - but who do not develop iGBS in the first 90 days of life. To account for the lower placental transfer associated with prematurity they should also be born at ≥ 34 weeks’ gestation ^16^. Additionally, they should not be born via caesarean section or have received IAP, as these will reduce their chance of developing EOD, and they should not have received a blood transfusion in the last month, as this may artificially affect their anti-GBS IgG concentrations.

We propose recruiting such controls through a prospective pregnancy swab study in which a rectovaginal swab will be obtained together with maternal delivery and cord sera during this feasibility study. The sample size for this study would be based on the need for a ratio of at least 3:1 controls per case for the main serotypes (III and Ia). In South-Western Uganda 28.8% of women are found to be GBS colonised ^17^. There is no serotypes distribution data. However, it is likely that serotypes will be similar to those in South Africa (32.8% III, 39.2% Ia) ^18^. Thus ~ 70 iGBS cases of ST III iGBS will require matching with 210 controls (women colonised with ST III) i.e. 210/0.328 ~ 640 carriers, which implies 640/0.288 = 2222 women to be swabbed in the pregnancy swab study (this feasibility study). We will double this number to account for caesarean section, premature birth and intrapartum antibiotic exposure.

### Study setting

The study is based at two Ugandan sites: one maternity national referral hospital (Kawempe Hospital, Kampala) and one general national referral hospital (Mulago Hospital, Kampala). Kawempe Hospital is the largest national referral hospital for pregnancies in Kampala, Uganda’s capital city, taking high-risk pregnancies from across the surrounding areas and all deliveries from the local community. There is a small neonatal unit admitting all infants weighing under 1000g as well as neonates with birth-related complications, sepsis or congenital anomalies (approximately 11,000 admissions per year). There are approximately 25,000 births per year at Kawempe. Mulago National Referral Hospital is a large hospital in Kampala with an official bed capacity of 1790. The hospital’s children’s services include outpatients, acute admissions and an inpatient malnutrition unit, as well as a weekly paediatric neurology clinic. The latter offers investigation and management of neurological conditions and a clinic-based occupational therapy and physiotherapy service for children with cerebral palsy and other Neurodevelopmental Impairments (NDIs).

### Types of participants, recruitment process and consent

The study has two entry points, women and their infants consenting at birth (birth cohort), and any infant with signs of infection presenting before they are 3 months old (active surveillance cohort).

For the Birth Cohort the study will be advertised via posters in antenatal clinic to assist with sensitisation and a film (available as extended data ^19^) will be produced to provide information to caregivers regarding neonatal sepsis and GBS. Pregnant women will be sensitised during antenatal clinic about the study and then approached at admission to the labour ward. All consenting women will be offered a rapid HIV and syphilis test in case they had not been tested in the current pregnancy. Women will be included if they are over the age of 18 years and delivering at Kawempe hospital or if they are emancipated minors aged between 14 and 17 years of age (live or stillbirth) and are willing to stay in the area for the first three months of life (or willing to travel to clinic until their child is 2 years old if their infant has known or presumed GBS infection).

For the Active surveillance cohort: all infants aged 0–90 days presenting to any of the paediatric units with signs and symptoms of sepsis will have a blood culture, lumbar puncture (if indicated) and nasal swab collected prior to receipt of antibiotics. Caregivers of any infant with culture-confirmed GBS infection will be approached for consent to follow up infants until they are two years old to assess their neurodevelopmental outcomes.

**Figure 1.**
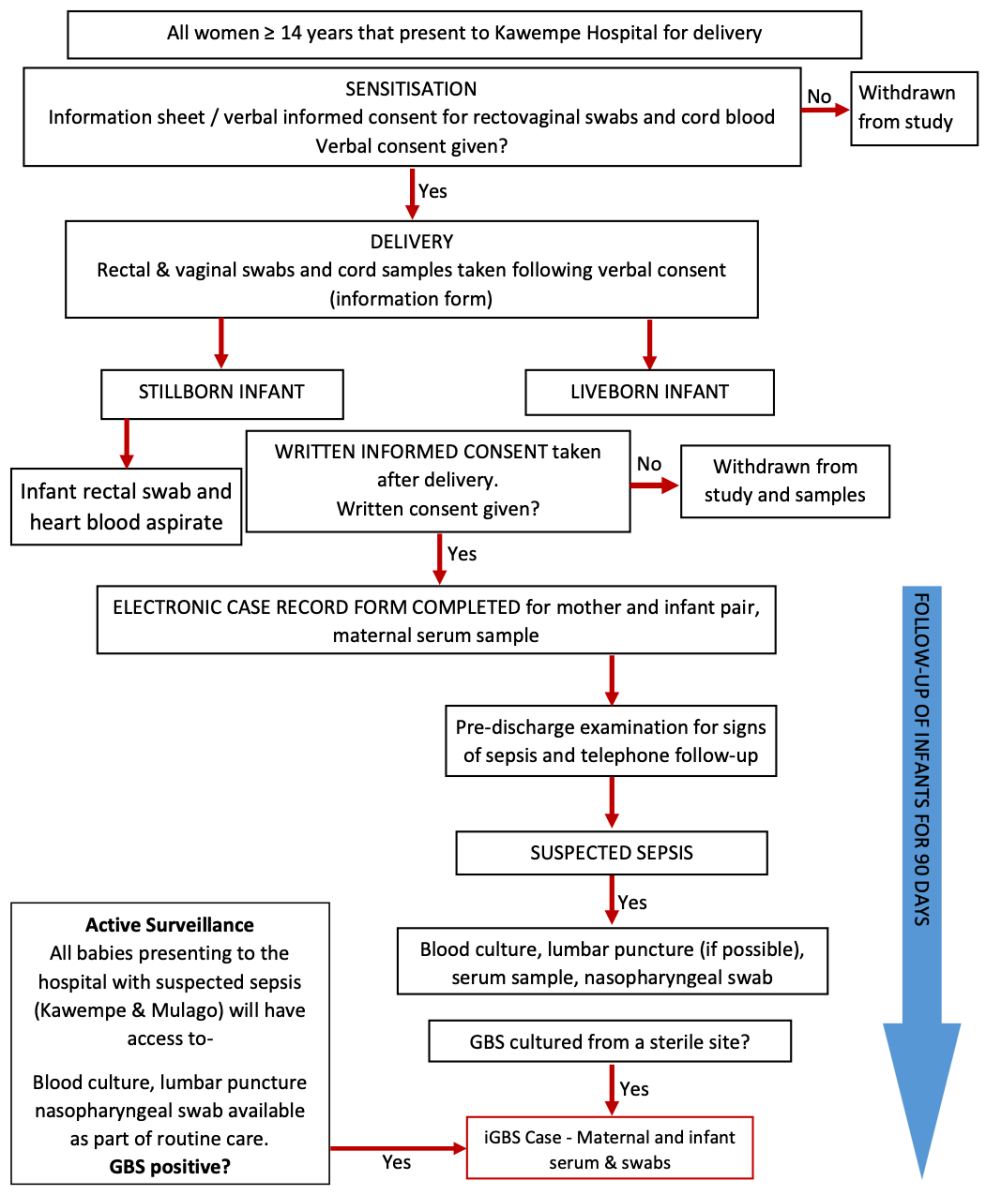
Participant Flow Diagram. GBS=Group B Streptococcus; iGBS=invasive GBS disease.

See Figure 1 for the study flow diagram.

### Identification of participant for qualitative research (focus group discussions and key informant interviews)

- *Community opinion leaders*: These will be drawn from the communities surrounding Mulago/Kawempe hospitals and will include – religious leaders of the Protestant, Catholic and Muslim denominations, local political leaders of the villages (local council chairpersons and the secretary for women affairs of the respective executive committees) and one representative of the village health team, where available.

- *Health service providers*: Midwives who attend to pregnant women in the antenatal clinic and those that administer immunizations to pregnant women.

- *Pregnant women*: Pregnant women who have been immunized during the current pregnancy will be approached for interview. They will be divided into the young (16 – 25 years) and the mature (>25 years) to avoid the mature women overshadowing the young ones during the discussions.

### Study evaluations of birth cohort dyad

All birth cohort mother-infant pairs will undergo a limited newborn and maternal examination on the postnatal ward prior to discharge to examine the baby for any external congenital abnormalities and signs and symptoms of maternal or neonatal infection. The mother-infant pairs are followed up after discharge through telephone calls conducted by a health visitor at day 3 of life (+/- 2 days), day 30 of life (+/- 7 days) and day 90 of life (+/- 14 days). If the day three phone call is unsuccessful, then a second call will be made of day 7 of life (+/- 3 days). All phone calls will be attempted on three occasions at different times of day if the first call is unsuccessful. Data recorded will include: the health status of mother and child; any medical care sought, any overnight hospital admissions; any courses of intravenous or oral antibiotic therapy; the presence or absence of any signs of neonatal sepsis. In addition, any mother-infant pair attending for Expanded Programme on Immunization (EPI) visits at 6 and 10 weeks of age will be reviewed by study staff to establish health status and signs of infection.

**Figure 2.**
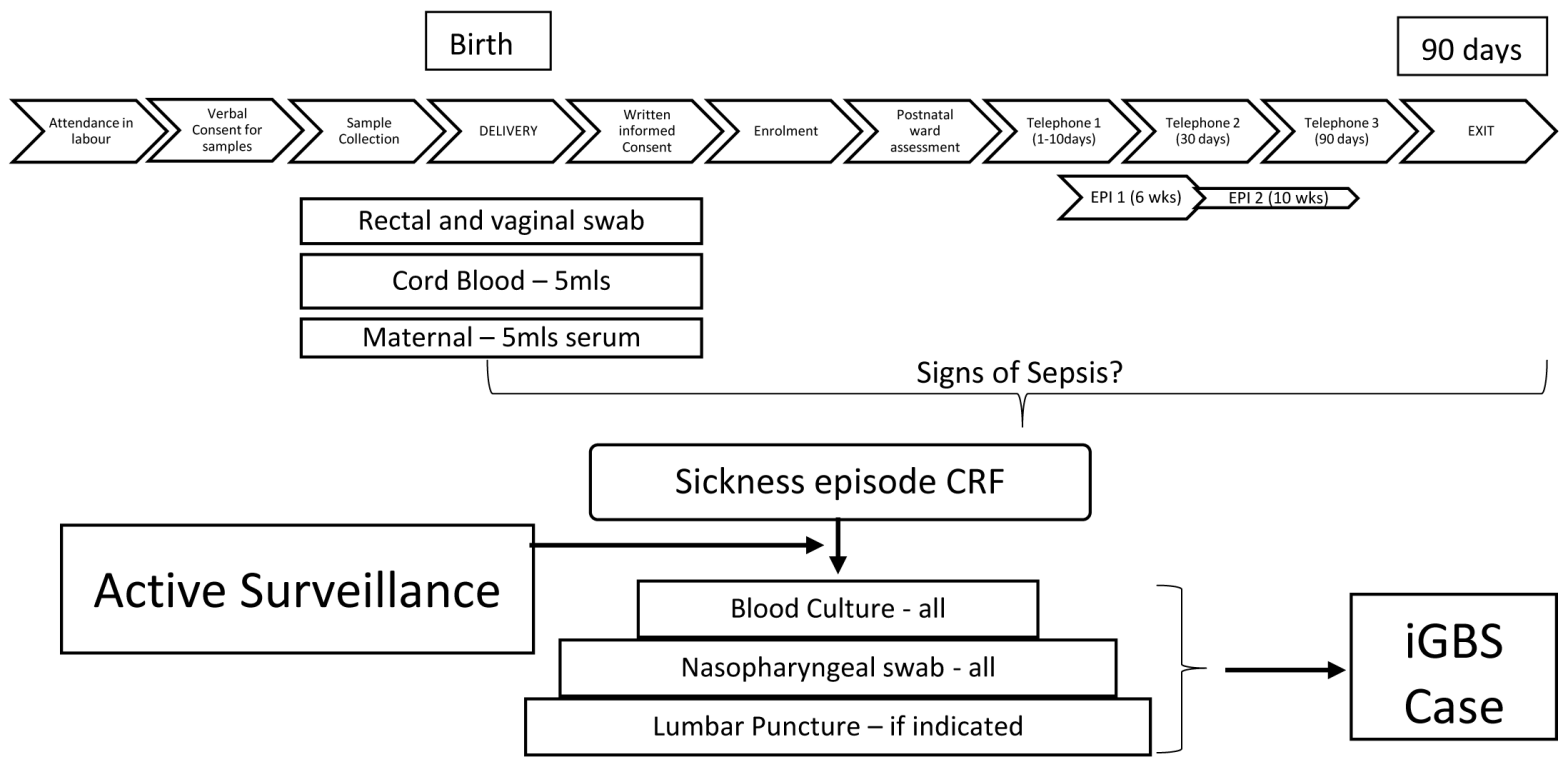
Birth Cohort Follow Up. NPS=nasopharyngeal swab; LP=lumbar puncture; GBS=Group B Streptococcus; HINE= Hammersmith Infant Neurological Assessment; OFC=occipital frontal circumference; BSID=Bayley Scales of Infant development; PEDI=Paediatric Evaluation of disability instrument; EPI= extended programme on immunisations; iGBS= invasive GBS disease.

**Figure 3.**
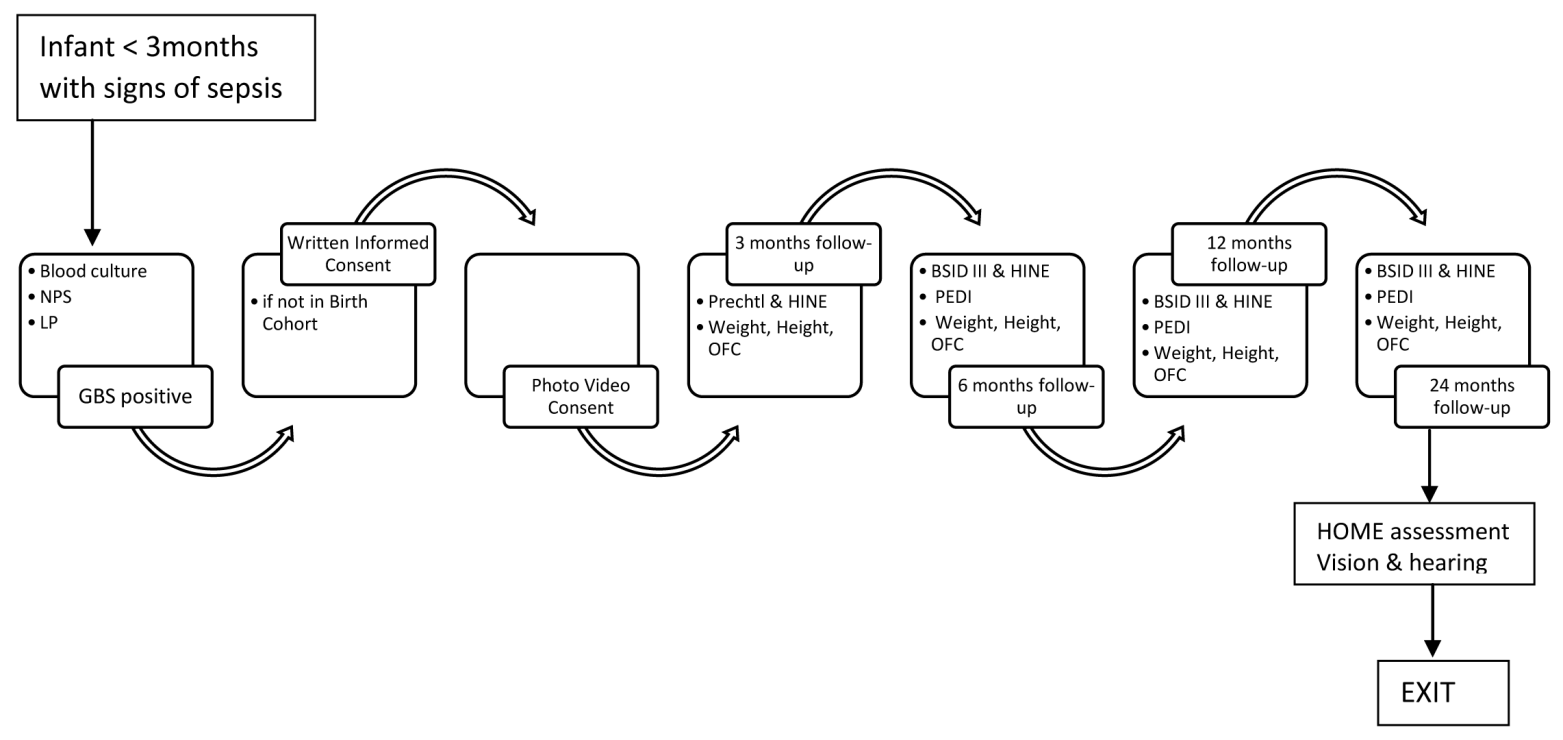
Neurodevelopmental Follow Up. NPS=nasopharyngeal swab; LP=lumbar puncture; GBS=Group B Streptococcus; HINE= Hammersmith Infant Neurological Assessment; OFC=occipital frontal circumference; BSID=Bayley Scales of Infant development; PEDI=Paediatric Evaluation of disability instrument; EPI= extended programme on immunisations; iGBS= invasive GBS disease.

During the three months of follow up any infant presenting with signs of sepsis will be examined by study staff and their clinical signs and investigations and any antibiotics administered will be recorded. Any infant with culture-confirmed GBS disease will be recruited into the neurodevelopmental follow up cohort. See Figure 2 and Figure 3 for details of follow up for both cohorts.

### Sample collection of birth cohort dyad

All women will be approached for verbal informed consent (available as extended data ^19^) at the time of admission in labour in order to collect a rectal swab, vaginal swab and cord blood at delivery. Full written informed consent will be obtained prior to discharge from hospital. If written consent is not obtained, samples will be destroyed.

Once full written informed consent (available as extended data ^19^) is obtained a maternal serum sample will be collected for anti-GBS antibody level and routine blood screening in pregnancy (HIV status, syphilis and hepatitis B). Any positive results will be reported back to the clinical team so that appropriate treatment can be initiated.

An assessment of the feasibility of performing minimally invasive tissue sampling on stillborn babies and rectal swabs from premature infants will be undertaken in order to finalise sample collection methodology for the main study.

### Study evaluations active surveillance cohort

Any liveborn infant that develops signs of infection between birth and 3 months of age will have a blood culture and lumbar puncture taken (if clinically indicated) and a nasopharyngeal swab. All infants will be reviewed on admission and information recorded on signs and symptoms, antimicrobial therapy and infant outcome (alive, died).

Any infant with culture-confirmed GBS disease will be recruited into the neurodevelopmental follow up cohort. A serum sample will be taken from mother and infant for cases of GBS disease, vagino-rectal swabs from the mother and rectal swab from the infant.

### Study evaluations in survivors of GBS infection (birth or active surveillance cohort)

All infants with GBS disease will be followed-up until the age of two years. A trained psychologist will carry out the assessments. We will compare the neurodevelopment of infants with GBS with normal healthy controls with a ratio of 1 case to 1 control. Prechtl, Bayley Scales of Infant Development III (BSID-III) and HINE assessments will be performed during neurodevelopmental follow-up if infants with GBS disease. *See Table 2* and Figure 3.

**Table 2.** Neurodevelopmental assessments of infants with GBS disease.

Age at follow-up	Neurodevelopment	Child functioning	Growth
3 months	Prechtl & HINE		Weight, Height, OFC
6 months	BSID III & HINE	PEDI	Weight, Height, OFC
12 months	BSID III & HINE	PEDI	Weight, Height, OFC
24 months	BSID III & HINE HOME assessment Vision & hearing	PEDI	Weight, Height, OFC

Legend - abbreviations: HINE= Hammersmith Infant Neurological Assessment; OFC=occipital frontal circumference; BSID=Bayley Scales of Infant Development; PEDI=Paediatric Evaluation of Disability Inventory.

Bayley Scales of Infant Development III (BSID-III) are used to assess neurodevelopment across five domains; receptive and expressive language, cognition, fine motor and gross motor according to standardised procedures. Bayley Scales have been validated in Uganda for use and provide a more detailed examination of neurodevelopmental domains than Ages and Stages (a screening tool). The HINE scores are video-recorded for quality control purposes. The recorded assessment are reviewed by another examiner to ensure consistency in the scoring ^20^.

### Focus group discussions, key informant and in-depth interviews

A series of focus group discussions, key informant and in-depth interviews will be conducted among service providers, program managers, community opinion leaders and pregnant women to explore ideas regarding sampling during labour, newborn sepsis and maternal immunization practices. Focus groups will contain 6-10 participants and will be conducted until saturation. Key informant interviews will be conducted with members of the focus group discussions to explore key themes identified. Focus Group Discussion guides are available as extended data ^19^.

### Laboratory evaluations

Rectal and vaginal swabs will be evaluated at MRC UVRI & LSHTM Research Unit, Entebbe, Uganda. Samples are sent to MRC with cold chain maintained at the end of each working day and plated on the same day. Maternal and infant swabs will be cultured in Todd Hewitt Broth and incubated for 24hrs before being sub-cultured on Chromagar Strep B ^®^. After 48hrs of incubation, presumptive GBS colonies will be identified by latex agglutination using StrepB® commercial kits and confirmed by rt-PCR and serotyped by gel-based PCR.

*Rt-PCR: For rt-PCR* primers as described in Diaz *et al.* (2010) will be used ^21^ and a TaqMan® probe. Primers and probes are outlined in Table 3. PCR will be performed using a LightCycler (Roche). The following PCR components will be added to each well in duplicate: 0.5 **m**M primer, 0.2 **m**M probe, 2.0 mM MgCl2, 0.05 mM dH2O (2 **μ**l template in a final volume of 50 **μ**l), 22.5μL Mastermix (Roche), 2.5mL sample DNA. A cycle threshold (Ct) value of 40 will be used as the cut-off for positive fluorescence detection signal of target amplification. Two positive controls and six negative controls will be included in all runs. Purified DNA from a known GBS strain will be used as positive control (in replicate) in a dilution corresponding to 10 ^7^ bacterial genomes. As a negative control, sterile water will be added instead of DNA template into each of the six negative control wells. Each isolate will be tested in duplicate.

*Gel-based PCR:* For gel-based PCR for GBS serotyping, primers and components described by Imperi *et al.* (2010) will be used and primers are outlined in Table 3. PCR will be performed on a thermocycler. The PCR products will be subsequently visualised on a 1.5% agarose gel.

**Table 3.** Sequences of primers and probes and cycling conditions for Real time and gel-based PCR.

PCR Target	GenBank accession number	Sequence of primers and probes	Cycling Conditions	Reference
*Cfb*	X72754	Primer 1: ATC CTG AGA CAA CAC TGA CA (position 263–282) Primer 2: TTG CTG GTG TTT CTA TTT TCA (position 340–320) TaqMan probe: 6-FAM–ATC AGA AGA GTC ATA CTG CCA CTT C–TAMRA (position 293–317)	50 °C (2 min); 95°C (10 mins); 45 cycles of 95°C (15 s), 60 °C (60 s)	Diaz 2013 ^21^
CpsI-Ia-6-7-F CpsI-6-R CpsI-7-R CpsI-F cpsL-R cpsG-F cpsG-R cpsG-2-3-6-R cpsN-5-F cpsN-5-R cpsJ-8-F cpsJ-8-R cpsJ-2-4-F cpsJ-2-R cpsJ-4-R cpsI-7-9-F cpsI-9-R cpsJ-Ib-F cpsJ-Ib-R	AB028896 AF337958 AB028896 AF163833 AB028896 AF163833 AB028896 AF163833 AB028896 AF337958 AB050723 AB050723 AF163833 AF349539 AB050723 AY375363 AF355776 AB050723 AY376403	GAATTGATAACTTTTGTGGATTGCGATGA CAATTCTGTCGGACTATCCTGATG TGTCGCTTCCACACTGAGTGTTGA CAATCCTAAGTATTTTCGGTTCATT TAGGAACATGTTCATTAACATAGC ACATGAACAGCAGTTCAACCGT ATGCTCTCCAAACTGTTCTTGT TCCATCTACATCTTCAATCCAAGC ATGCAACCAAGTGATTATCATGTA CTCTTCACTCTTTAGTGTAGGTAT TATTTGGGAGGTAATCAAGAGACA GTTTGGAGCATTCAAGATAACTCT CATTTATTGATTCAGACGATTACATTGA CCTCTTTCTCTAAAATATTCCAACC CCTCAGGATATTTACGAATTCTGTA CTGTAATTGGAGGAATGTGGATCG AATCATCTTCATAATTTATCTCCCATT GCAATTCTTAACAGAATATTCAGTTG GCGTTTCTTTATCACATACTCTTG	95 °C (15 min); 35 cycles of 95°C (60 s), 54 °C (60 s), 72°C (2 min); 72°C (10 min).	Imperi 2010 ^22^

Pure GBS confirmed isolates will be stored on microbeads at -80˚C.

Blood and cerebrospinal fluid cultures will be evaluated at Makerere Microbiology Laboratories, Kampala from stillborn infants and blood cultures from infants with signs and symptoms of infection. All blood cultures are analysed within 12 hours of collection. At least 1mL of blood will be collected in BACTEC bottles and incubated for 36 hours (10). Cell count, gram stain and culture will be performed on any cerebrospinal fluid. Any presumptive positive culture will be plated onto selective agar and identified by CAMP test. Any Pure GBS isolates will be stored on microbeads at -80˚C.

Cord and maternal/infant blood samples will be evaluated at MRC/UVRI at LSHTM Entebbe Uganda. The serum obtained will be analysed for antibody concentration using a standardized multiplex Luminex assay still under development and antibody function using an opsonophagocytic killing assay using standard reagents developed as part of the Bill and Melinda Gates funded assay standardization project (OPP1153630) to determine antibody concentrations for infants with and without invasive GBS disease. Maternal serum will be tested for HIV, syphilis and hepatitis B.

### Primary outcomes

Maternal anti-GBS antibody concentration in infants with GBS disease compared to healthy controls.

Ability to recruit at least 50% of women delivering and collect samples

Acceptability of the study i.e. acceptability of sampling for live and stillbirths and infants with infections

### Secondary outcomes

- serotype-specific GBS colonisation rates during pregnancy in Kampala

- neonatal GBS disease rates

- GBS-related neurological impairment rates

- GBS –associated stillbirth rates

- all cause neonatal infection rates

### Data entry, analysis and quality assurance

Detailed demographic data from pregnant woman and infant demographic information will be recorded and managed through the use of REDCap (Research Electronic Data Capture) ^23^, a secure web-based electronic data collection platform. The REDCap server is hosted by Makerere University John’s Hopkins University, in Kampala. Audio recordings are used to collect all qualitative study data. These are then transcribed to Microsoft Word and analyzed using themes-based analysis.

Once captured, all quantitative data is subjected to cleaning and quality assurance processes before it is analysed. The PROGRESS GBS REDCap system has range, validation, and consistency checks to minimize data entry errors. Data reviews are undertaken at three stages, on day of collection by study coordinator, within one week by dedicated quality assurance technicians and weekly by the data manager to minimise any errors. Password protection for individual users is present on all electronic data entry systems.

Data collected includes:mode of delivery, antenatal risk factors for infection (Premature rupture of membranes/chorioamnionitis/maternal fever/raised white cell count), number of live births, information on maternal vaccination status, information of any infections leading up to delivery requiring antibiotics, time in hours of rupture of membranes prior to delivery, gestational age, birth weight, stillbirths/neonatal deaths/perinatal complications (birth asphyxia/meconium staining/signs of severe infection in the first three months of life (fever >38.5 degrees, raised white cell count, meningism). Gestational age calculated by ultrasonography if available; last normal menstrual period/ midwife palpation if ultrasound unavailable; Ballard score will also be performed on the newborn infants.

For any infants with suspected infection data is collected on maternal age, severity of infant illness (defined as need for hospital/intensive care stay), antimicrobial therapy and clinical outcome following disease (alive well, alive neurological impairment, death). For any infant with culture-confirmed GBS disease additional data is collected on pregnancy and birth as above. Data collection forms are available as extended data ^19^.

### Feasibility information

In order to determine the feasibility and acceptability of running a large sero-epidemiological study in this cohort, we will additionally collect information on enrollment rates, blood culture collection information (volume of blood culture, culture bottle weight, time of collection, time to BACTEC, time to positivity, time to Gram stain, time to final result, reporting of final result including antibiotic resistance, bacterial yield), ability to collect blood and CSF culture from infants with suspected sepsis and establish a denominator for infection rates (available as extended data ^19^). In addition, we will assess the feasibility and acceptability of our sampling methods. Monthly vertical audits of data and samples are undertaken by the quality assurance team.

Graphpad Prism version 8 will be used for antibody analysis. Geometric mean and median antibody titres will be calculated for cases and controls and comparisons made by t-test or Kruskall Wallis. reverse cumulative distribution curves and histogram plots will also be generated to compare antibody distribution between groups. Odds ratios for disease at different thresholds will then be calculated. Finally, plots will be constructed to demonstrate the probability of infection at different thresholds based on the known disease incidence. We anticipate that the antibody concentration will not be normally distributed and so will log transform the data. If the data are then normally distributed, we will undertake comparison of the geometric mean antibody concentration and if the data remains non-normal we will use the Kruskall Wallis tests of medians to compare antibody concentrations between groups.

Rates for colonisation, disease, all-cause and GBS infection, death and stillbirth, enrollment and retention will be expressed as % or per 1000 pregnancies/livebirths as appropriate.

### Ethical considerations

This study is conducted in accordance with the principles set forth in the ICH Harmonised Tripartite Guideline for Good Clinical Practice and the Declaration of Helsinki in its current version. The study has been approved by the Ugandan National Council for Science and Technology (UNCST; ref. HS 2496), Makerere University School of Medicine Research & Ethics Committee (SOMREC; 2018–130), and St George’s, University of London (SGUL REC ref 2020.0024) who sponsored the study.

### Dissemination of information

Data will be reported using the STROBE_IN guidelines for cohort studies https://www.strobe-statement.org/index.php?id=available-checklists, with the checklist deposited in OSF. Data will be disseminated through presentations to local and national bodies in Uganda and at international conferences. The final report will be prepared as a manuscript for publication.

### Study status

The PROGRESS GBS study commenced on 24 April 2019. The study was paused on 26 March 2020 due to the coronavirus pandemic and will restart on August 2020. The study successfully recruited 6000 participants between 24 April 2019 and 26 March 2020 and took blood cultures from 5527 infants with signs and symptoms of infection. The study is currently undertaking data cleaning in anticipation of the restart.

## Discussion

This feasibility clinical observational and qualitative study will provide unique evidence about the feasibility of undertaking sero-epidemiological studies in Uganda. The clinical cohort study will provide evidence for GBS colonization, GBS disease and association with stillbirth for the first time in Uganda. The follow-up of infants with GBS infection will provide evidence on neurodevelopmental outcomes in this group in a low-resource setting. Additionally, the study will provide evidence of pathogens and outcomes for 11,000 sepsis episodes in Uganda. Together, our results will contribute to global efforts to improve pregnancy, birth and neonatal infectious outcomes through maternal vaccination in Uganda. 

The study’s data collection is anticipated to be completed by October 2020 after which dissemination of the most important and relevant program learnings will begin at the end of 2020.

We directly developed the study design and conduct through engaging caregivers and programme facilitators (‘expert parents’). Through focus group discussions at a key-stakeholders meeting their priorities and were identified ^24^ and these contributed to our research question and outcome measures’ development. Engaging the local community is key to the success of this and any future studies to improve maternal and infant health and we continue to work with participants and the wider community with results as they are available.

There are strong links between our programme and partnership organisations working in Maternal and Child Health programming such as Kampala Capital City District Health Office and other collaborating institutions. In order to inform local and national health policies, we will disseminate research findings to the Ministry of Health. In a bid to contribute to the sustainability of the innovation at local and district levels, regional-level stakeholders, such as the District Health Office and heads of regional health and social services, will be engaged to support staff recruitment. In order to promote buy-in, facilitate fast-cycle learning, disseminate study findings and ultimately promote sustainability of the programme, meetings for key stakeholders, such as local Non-Governmental Organisations working in maternal and child health will be held at least once during the project period.

Lessons learned from this feasibility study will be key to informing the ability of a low-resource country like Uganda to equitably take part on sero-epidemiological studies and provide much needed data on disease burden and potential mitigations, such as maternal vaccination. Ways to minimize loss to follow-up in these settings during this vulnerable period of time; maximizing blood culture positivity or maintaining sample integrity and tracking from large cohorts that are geographically distant are some of the lessons anticipated.

## Data availability

### Underlying data

No data are associated with this article.

### Extended data

Open Science Framework: PROGRESS GBS. https://doi.org/10.17605/OSF.IO/5A86F ^19^

This project contains the following extended data:

– GBS Study Supplementary File 1_Denominator Data - Deliveries.docx
– GBS Study Supplementary File 2_Denominator Data – Infant admissions.docx
– GBS Study Supplementary File 3 - Data Collection Form.pdf
– GBS Study Supplementary File 4 - FGD guides.docx
– GBS Study Supplementary File 5 - Parent Information Leaflets and Consent Forms.docx
– GBS Study Supplementary File 6 - Film.mp4

Data are available under the terms of the Creative Commons Attribution 4.0 International license (CC-BY 4.0).
