# Supplementary material for: Seroepidemiology of maternally-derived antibody against Group B Streptococcus (GBS) in Mulago/Kawempe Hospitals Uganda - PROGRESS GBS
Source: Gates Open Res. 2020 Nov 13;4:155. Originally published 2020 Oct 8. [Version 2] doi: 10.12688/gatesopenres.13183.2 (PMC7706450; doi:10.12688/gatesopenres.13183.2)
Supplement: Peer review history

# Peer review: Reviewer response for version 2

**DOI:** 10.21956/gatesopenres.14415.r29983

**Published:** 2020-11-30

**Authors:** Marleen Temmerman

**Affiliations:** 1 Centre of Excellence in Women and Child Health, Department of Obstetrics and Gynecology, Aga Khan University, Nairobi, Kenya

The revisions are OK and I hereby approve the manuscript.

Is the study design appropriate for the research question?

Yes

Is the rationale for, and objectives of, the study clearly described?

Yes

Are sufficient details of the methods provided to allow replication by others?

Yes

Are the datasets clearly presented in a useable and accessible format?

Yes

Reviewer Expertise:

NA

I confirm that I have read this submission and believe that I have an appropriate level of expertise to confirm that it is of an acceptable scientific standard.

# Peer review: Reviewer response for version 2

**DOI:** 10.21956/gatesopenres.14415.r29982

**Published:** 2020-11-18

**Authors:** Chisomo L. Msefula

**Affiliations:** 1 Pathology Department, College of Medicine, University of Malawi, Blantyre, Malawi

The authors have adequately addressed all the comments.

Is the study design appropriate for the research question?

Yes

Is the rationale for, and objectives of, the study clearly described?

Yes

Are sufficient details of the methods provided to allow replication by others?

Yes

Are the datasets clearly presented in a useable and accessible format?

Not applicable

Reviewer Expertise:

Medical microbiology; Molecular microbiology; Diagnostics

I confirm that I have read this submission and believe that I have an appropriate level of expertise to confirm that it is of an acceptable scientific standard.

# Peer review: Reviewer response for version 1

**DOI:** 10.21956/gatesopenres.14387.r29759

**Published:** 2020-11-09

**Authors:** Marleen Temmerman

**Affiliations:** 1 Centre of Excellence in Women and Child Health, Department of Obstetrics and Gynecology, Aga Khan University, Nairobi, Kenya

Interesting study addressing an important public health/child health problem globally and in particular in LMIC, proposing a large sample size efficacy study needed to develop a maternal GBS vaccine. The proposal is well developed and well written, and I have only a few questions/comments:

1. Informed consent: a rectovaginal swab will be obtained together with maternal delivery and cord sera. Women will be asked for verbal informed consent during labour, which is to be avoided. This issue is addressed by asking written informed consent prior to discharge which is difficult for the woken to refuse.
  As sensitization for the study takes place during antenatal care, one could envisage to collect the rectovaginal swabs during late trimester.
2. HIV, syphilis and hepatitis samples have to be taken during pregnancy, not during labour.

Is the study design appropriate for the research question?

Yes

Is the rationale for, and objectives of, the study clearly described?

Yes

Are sufficient details of the methods provided to allow replication by others?

Yes

Are the datasets clearly presented in a useable and accessible format?

Yes

Reviewer Expertise:

OBS/GYN

I confirm that I have read this submission and believe that I have an appropriate level of expertise to confirm that it is of an acceptable scientific standard, however I have significant reservations, as outlined above.

# Peer review: Reviewer response for version 1

**DOI:** 10.21956/gatesopenres.14387.r29794

**Published:** 2020-10-29

**Authors:** Chisomo L. Msefula

**Affiliations:** 1 Pathology Department, College of Medicine, University of Malawi, Blantyre, Malawi

**Summary of planned study:**

 The study addresses the burden of Group B Streptococcus (GBS) in neonates and the potential for maternal antibodies as a sero-correlate of protection. The aim is to evaluate the feasibility and acceptability of a GBS sero-epidemiology study, in Kampala, Uganda, a resource limited setting. Anti-GBS antibody titres will be assessed in infants with GBS infection compared to healthy controls. A mother and her infant will be recruited into the study at birth, and also any infant less than 3 years old with signs of infection will be recruited. Infants recruited at birth will be followed for 90 days. Any infant with confirmed GBS infection will be followed for two years during which a psychologist will assess neurodevelopment. Rectal and vaginal swabs will be collected from the mothers for GBS growth, and also cord blood at delivery. Blood culture will be done on blood from infants with suspected infection but also from stillbirths. A qualitative study employing focus group discussions, key informant and in-depth interviews will be nested in the project and the aim is to explore ideas regarding sampling during labour, newborn sepsis and maternal immunization practices. Secondary data such as ways to minimize loss to follow-up in a  resource limited setting; maximizing blood culture positivity or maintaining sample integrity and tracking from large cohorts that are geographically distant, will also be collected.

**My comments on the protocol:**

- In Figure 1, the Flow diagram, there is a typo error in Active surveillance box.
- Considering that HIV impacts on GBS maternal antibody levels (Le Doare *et al.*, 2015 ^1^), What happens when the mother is found HIV-infected? Do they proceed in the study? What is the analysis plan for HIV-infected mothers and or HIV-exposed infants? This study is located in an HIV endemic region and you might need to mention in your background the impact of HIV on maternal antibodies.
- Sample size calculation has been well considered and calculated. Should you also have considered the HIV rates?
- Your participant flow chart indicates recruitment from 14 years old. Why are you excluding mothers aged 14 to 15 years old in the focus group discussions? How about men (husbands or partners of pregnant women? (Merten *et al.*, 2015 ^2^).
- You have presented the sentences below taken from qPCR section as a study that has already taken place, and not a study protocol. Please correct to future tense. “…………. Two positive controls and six negative controls were included in all runs. Purified DNA from a known GBS strain was used as positive control in a dilution corresponding to 10 ^7^ bacterial genomes. As a negative control, sterile water was added instead of DNA template. Each isolate was tested in duplicate……”.
- PCR section - You have specified one positive control of the mentioned two and one negative control of the six. What are the others? Or do you mean there will be 2 replicates of the positive control and six replicates of the negative control?
- The manuscript refers to components of mastermix as PCR conditions in the narrative (PCR section), but the cycling conditions have been rightly listed in Table 3.
- The PCR narrative refers to real-time PCR. Why is Table 3 providing cycling conditions for gel-based PCR? Also, the narrative has specified only one PCR target, *CFB, *but Table 3 has multiple other targets. Can you please clarify?
- Samples will be processed at MRC and Makerere. How far are these laboratories from the sample collection sites? You might consider including in your methods how the samples will be transferred and under what conditions, to maintain integrity.

Is the study design appropriate for the research question?

Yes

Is the rationale for, and objectives of, the study clearly described?

Yes

Are sufficient details of the methods provided to allow replication by others?

Yes

Are the datasets clearly presented in a useable and accessible format?

Not applicable

Reviewer Expertise:

Medical microbiology; Molecular microbiology; Diagnostics

I confirm that I have read this submission and believe that I have an appropriate level of expertise to confirm that it is of an acceptable scientific standard, however I have significant reservations, as outlined above.

## References

[1] : Anti-group B Streptococcus antibody in infants born to mothers with human immunodeficiency virus (HIV) infection. *Vaccine*.2015;33(5) : 10.1016/j.vaccine.2014.12.025 621-7 10.1016/j.vaccine.2014.12.025 25543061PMC4315133

[2] : Gender Determinants of Vaccination Status in Children: Evidence from a Meta-Ethnographic Systematic Review. *PLoS One*.2015;10(8) : 10.1371/journal.pone.0135222 e0135222 10.1371/journal.pone.0135222 26317975PMC4552892

# Response

**Published:** 2020-11-03

**Authors:** kirsty le doare

Many thanks for your review of our protocol. We have taken note of your comments and amended the article as you suggest:

- *In Figure 1, the Flow diagram, there is a typo error in Active surveillance box.*
**This is now corrected and a new version uploaded.**
- *Considering that HIV impacts on GBS maternal antibody levels (Le Doare et al., 2015* *^1^* *), What happens when the mother is found HIV-infected? Do they proceed in the study? What is the analysis plan for HIV-infected mothers and or HIV-exposed infants? This study is located in an HIV endemic region and you might need to mention in your background the impact of HIV on maternal antibodies.*
**We are recruiting all mothers, whether living with HIV or not. We will analyse risk of GBS disease in this cohort separately. We have added some additional background to our introduction as you suggest.**
- *Sample size calculation has been well considered and calculated. Should you also have considered the HIV rates?*
**As this is a feasibility study, we will collect information on all women and then undertake a sensitivity analysis by HIV status.**
- *Your participant flow chart indicates recruitment from 14 years old. Why are you excluding mothers aged 14 to 15 years old in the focus group discussions? How about men (husbands or partners of pregnant women? (Merten et al., 2015* *^2^* *).*
**The focus groups include pregnant women, men, community leaders and other key influencers. Because of cultural sensitivities, we have not included pregnant women <16 years.**
- *You have presented the sentences below taken from qPCR section as a study that has already taken place, and not a study protocol. Please correct to future tense. “…………. Two positive controls and six negative controls were included in all runs. Purified DNA from a known GBS strain was used as positive control in a dilution corresponding to 10 ^7^ bacterial genomes. As a negative control, sterile water was added instead of DNA template. Each isolate was tested in duplicate……”.*

**Thank you for noticing this error, we have now corrected to future tense.**

- *PCR section – You have specified one positive control of the mentioned two and one negative control of the six. What are the others? Or do you mean there will be 2 replicates of the positive control and six replicates of the negative control?*

**As you correctly identify, we will have replicates of the positive and negative controls.**

- *The manuscript refers to components of mastermix as PCR conditions in the narrative (PCR section), but the cycling conditions have been rightly listed in Table 3.*

**The mastermix referred to in the narrative is the platinum mastermix to which additional reagents are added. As such, we believe that the description is correct. We have changed the text to clarify this.**

- *The PCR narrative refers to real-time PCR. Why is Table 3 providing cycling conditions for gel-based PCR? Also, the narrative has specified only one PCR target, CFB, but Table 3 has multiple other targets. Can you please clarify?*

**Thank you for pointing this out. The serotyping PCR is a gel-based PCR, this has now been clarified in the text. The *cfb* gene was used to identify GBS by rt-PCR but serotyping has multiple targets as you point out. We have clarified this in the text.**

- *Samples will be processed at MRC and Makerere. How far are these laboratories from the sample collection sites? You might consider including in your methods how the samples will be transferred and under what conditions, to maintain integrity.*

**The blood cultures will be taken to Makarere University for processing, this is approximately 4km from the trial site. We have spent a considerable amount of effort to ensure that sample integrity is maintained. Blood cultures arrive for culture within 12 hours of collection. Swabs will be transferred to MRC daily with cold chain maintained and cultured on the same day. Centrifuged blood samples will be transferred daily to MRC and cultured will be aliquoted and frozen. We have added this to the protocol.**
